# In vitro Antibiotic and Modulatory Activity of *Mesosphaerum suaveolens* (L.) Kuntze against *Candida* strains

**DOI:** 10.3390/antibiotics9020046

**Published:** 2020-01-27

**Authors:** Adrielle Rodrigues Costa, José Weverton Almeida Bezerra, Rafael Pereira da Cruz, Maria Audilene de Freitas, Viviane Bezerra da Silva, João Cruz Neto, Antonia Thassya Lucas dos Santos, Maria Flaviana Bezerra Morais Braga, Leomara Andrade da Silva, Maria Ivaneide Rocha, Jean Paul Kamdem, Marcello Iriti, Sara Vitalini, Antonia Eliene Duarte, Luiz Marivando Barros

**Affiliations:** 1 Postgraduate Program in Molecular Bioprospecting, Regional University of Cariri (URCA), Crato 63122-290, CE, Brazil; adrielle.arc@hotmail.com (A.R.C.); viviane123silvab@gmail.com (V.B.d.S.); 2 Postgraduate Program of Plant Biology, Federal University of Pernambuco, Recife 50670-901, Brazil; weverton.almeida@urca.br; 3 Laboratory of Applied Mycology of Cariri, Regional University of Cariri (URCA), Crato 63122-290, CE, Brazil; rafaelcruz284@gmail.com (R.P.d.C.); audbiologa@hotmail.com (M.A.d.F.); thassyalucas@hotmail.com (A.T.L.d.S.); 4 Nursing Course at the Regional University of Cariri (URCA), Crato-CE-Brazil; enfjcncruz@gmail.com; 5 Postgraduate Program in Botany – National Amazon Research Institute (INPA), Manaus 69067-375, AM, Brazil; andrade.biologia@hotmail.com; 6 Biology and Toxicology Laboratory, University of Regional Cariri (URCA), Crato 63122-290, CE, Brazilkamdemjeanpaul2005@yahoo.fr (J.P.K.); duarte105@yahoo.com.br (A.E.D.); 7 Department of Agricultural and Environmental Sciences, Milan State University, 20133 Milan, Italy; marcello.iriti@unimi.it; 8 Vegetable Ecophysiology Laboratory, Regional University of Cariri, Crato 63122-290, CE, Brazil; lmarivando@hotmail.com

**Keywords:** *Hyptis suaveolens*, *Candida*, HPLC-DAD, bamburral, phychochemistry

## Abstract

The emergence of fungal resistance to commercial drugs has been a major problem for the WHO. In this context, research with natural products is promising in the discovery of new active substances. Thus, this work evaluated the antifungal effect of a medicinal plant (i.e., *Mesosphaerum suaveolens*) against strains of the genus *Candida*, tested the combined effect with the drug fluconazole, and, finally, determined the phenolic constituents present in the species. Initially, aqueous extracts of leaves (AELMs) and aerial parts (AEAPMs) of the species were prepared. For microbiological assays, the minimum fungicidal concentration was determined by broth microdilution, and the combined effect of fluconazole extracts were verified by sub-inhibitory microdilution concentrations (CFM/8) followed by spectrophotometric readings which were used to determine the IC_50_. HPLC detected the presence of flavonoids and phenolic acids, detecting eight compounds present in the samples of which caffeic acid and quercetin were major components. The AELMs modulated fluconazole activity since it decreased fluconazole’s IC_50_ from 7.8 µg/mL to an IC_50_ of 4.7 µg/mL (CA LM 77) and from 28.8 µg/mL to 18.26 µg/mL (CA INCQS 40006) for the *C. albicans* strains. The AEAPMs were able to potentiate the effect of fluconazole more effectively than the AELMs. Such an effect was significant for the 16 µg/mL concentration for CA LM 77 and 32 µg/mL for CA INCQS 40006. The AEAPMs as well as the AELMs presented clinically relevant activities for *C. tropicalis* strains. For the *C. tropicalis* LM 23 strain, the AEPMs obtained an IC_50_ of 25 µg/mL and the AELMs an IC_50_ of 359.9 µg/mL.

## 1. Introduction

*Candida* yeasts reside in humans as commensals and are part of the normal microbiota in healthy subjects. When an imbalance between the microbiota and the host’s immune system occurs, these fungi can become pathogenic, causing candidiasis and presenting different clinical forms depending on the type of infection and the degree of immunosuppression. Nevertheless, mucosal, systemic, and allergic skin reactions are the common clinical presentation [1,2,3,4].

Infections caused by *Candida* yeasts are related to a high morbidity and mortality rate in which these species are caused by superficial and systemic candidiasis, and the latter represents a serious problem for health systems and patients [5].

In addition to *Candida albicans*, other species are considered candidiasis agents such as *Candida glabrata*, *Candida tropicalis*, *Candida krusei*, and *Candida parapsilopsis* [6]. *Candida* species cause whitish, lumpy, odorless, and non-purulent vaginal discharge [7]; other symptoms include itching, burning, redness, odorless discharge, injury, pain, edema, erythema, interdigital erosion, folliculitis, exudate, purulence, onychomycosis, and paronychia [8,9,10,11].

Associated with such problems is fungal resistance which represents a major clinical challenge in the treatment of invasive infections due to the scarcity of effective antifungals available. In addition, current drugs may be limited by drug actions and severe adverse effects [12].

The indiscriminate use of antifungal drugs favors an increase in microbial resistance; thus, we seek to understand the mechanisms of antifungal drugs and the molecular cellular mechanisms involved in the process of antifungal resistance [13]. Due to the limited number of commercially available antifungal agents and their various side effects, there is a need to produce new and more efficient antifungal agents with few adverse effects [14].

In response to this problem, research focusing on plant bioprospecting has been expanding in Brazil and worldwide, thereby increasing the importance and knowledge about their chemical constituents [15]. In this sense, studies for new antifungal agents acquired based on extracts, fractions, and essential oils or isolated constituents of plants from Brazilian flora have intensified, seeking to reverse the resistance of *Candida* spp. [16,17].

Recently, many plants have been evaluated not only for their antifungal activity but also as modifiers of antibiotic resistance [18], since plant-derived natural products are rich in phytochemicals, such as polyphenols, which are known to exhibit a variety of pharmacological activities [19,20]. In addition, polyphenols can act directly on the microbiological membrane, causing irrecoverable damage [21]. Caffeic acid is of particular importance, as it is present in *M. suaveolens* extracts which have been reported to be relevant in combating microbiological resistance to first-line drugs [22,23,24,25].

According to Batista et al. [26] and Castro et al. [27], microbiological resistance mechanisms usually occur during treatment, this being the reason prolonged exposure to drugs favors fungal resistance. In this context, the search for alternative treatments have increased and one important aspect to consider has been the popular use of plant extracts by local communities to cure or ameliorate symptoms associated with infectious diseases. It should also be stressed that plant extracts are regarded by the population as safe and free of side effects. 

*Mesosphaerum suaveolens* (L.) Kuntze, which belongs to the Lamiaceae family, is native to the Americas and is commonly known in Brazil as “bamburral”" or “alfazema-brava” [28]. *Mesosphaerum suaveolens* is used in traditional Brazilian medicine as a carminative as well as for the treatment of stomach pain and inflammation [29,30]. Substantial evidence from the literature reveals that *M. suaveolens* exhibits a variety of pharmacological activities including anti-inflammatory [30,31], insecticidal [22,32], larvicidal [33], and antioxidant [23].

Given the biological activities of *M. suaveolens*, especially with respect to its antimicrobial potential, it was hypothesized that *M. suaveolens* may afford protection against different *Candida* strains. Therefore, the objective of this study was to evaluate the antifungal activity and modulatory potential of *M. suaveolens* extracts against yeast from the *Candida* genus as well as to determine the phenolic compounds present in the extracts.

## 2. Results

### 2.1. Phytochemical Analysis 

The HPLC fingerprinting composition of *M. suaveolens* extract revealed the presence of gallic acid (retention time − t_R_ = 9.73 min; peak 1), catechin (t_R_ = 14.96 min; peak 2), chlorogenic acid (t_R_ = 20.58 min; peak 3), caffeic acid (t_R_ = 24.07 min; peak 4), ellagic acid (t_R_ = 31.49 min; peak 5), rutin (t_R_ = 40.13 min; peak 6), quercetin (t_R_ = 46.93 min; peak 7) and apigenin (t_R_ = 65.11 min; peak 8). The respective retention times were compared to analytical standards and the substances present in *M. suaveolens* were identified.

**Figure 1:**
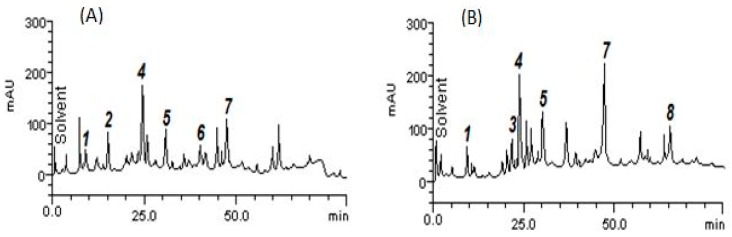
Representative sample of reverse phase HPLC analysis of the *Mesosphaerum suaveolens* extracts: (**a**) chromatogram of the *Mesosphaerum suaveolens* aqueous leaf extract (AELMs) showing its peaks; (**b**) chromatogram of the *Mesosphaerum suaveolens* aqueous aerial parts extract (AEAPMs) showing its peaks. Gallic acid (peak 1), catechin (peak 2), chlorogenic acid (peak 3), caffeic acid (peak 4), ellagic acid (peak 5), rutin (peak 6), quercetin (peak 7), and apigenin (peak 8).

The chemical characterization of the M. suaveolens leaf extract (A) and aerial parts extract (B) is depicted in Figure 1 and their quantification is provided in Table 1, where slight differences in their composition can be observed. Among the eight identified compounds, chlorogenic acid (peak 3) and apigenin (peak 8) were not found in the M. suaveolens aqueous leaf extract (Figure 1a, Table 1), while catechin (peak 2) and rutin (peak 6) were not identified in the M. suaveolens aqueous aerial parts extract (Figure 1B, Table 1). However, the chromatogram revealed the presence of gallic acid, caffeic acid, ellagic acid, and quercetin in both extracts at different quantities (Table 1). Peak 4: caffeic acid (AELMs: 13.27 mg/g and AEAPMs: 14.25 mg/g) and peak 7: quercetin (AELMs: 3.7 mg/g and AEAPMs: 14.70 mg/g) were more significant in both extracts.

### 2.2. Microbiological Activity

**Figure 2:**
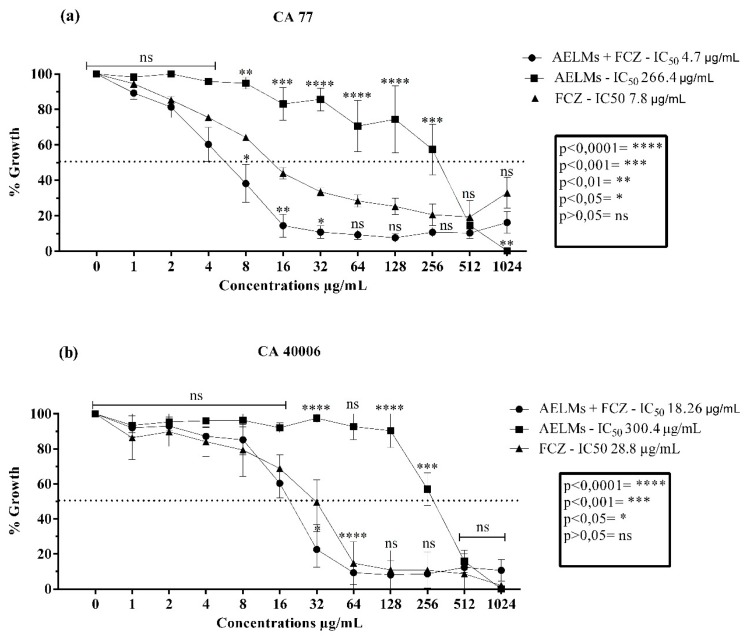
Cell viability curves and IC_50_ values (concentration responsible for 50% colony growth inhibition) of the *Mesosphaerum suaveolens* aqueous leaf extract (AELMs) and fluconazole (FCZ) in μg/mL against *Candida albicans*. (**a**) Antifungal activity against strains of *Candida albicans* LM 77. (**b**) Antifungal activity against strains of *Candida albicans* INCQS 77.

The *M. suaveolens* extracts showed antifungal activity against the *Candida* strains evaluated in this study. The leaf extract modulated fluconazole activity, since it decreased fluconazole’s IC_50_ from 7.8 µg/mL to an IC_50_ of 4.7 µg/mL (CA LM 77) and from 28.8 µg/mL to 18.26 µg/mL (CA INCQS 40006) for the *C. albicans* strains (Figure 2). Although this extract did not present an antifungal effect more potent than the standard drug, it presented anti-*Candida* action against the aforementioned strains, obtaining clinically relevant IC_50_ values of 266.5 µg/mL (CA LM 77) and 300.4 µg/mL (CA INCQS 40006).

**Figure 3:**
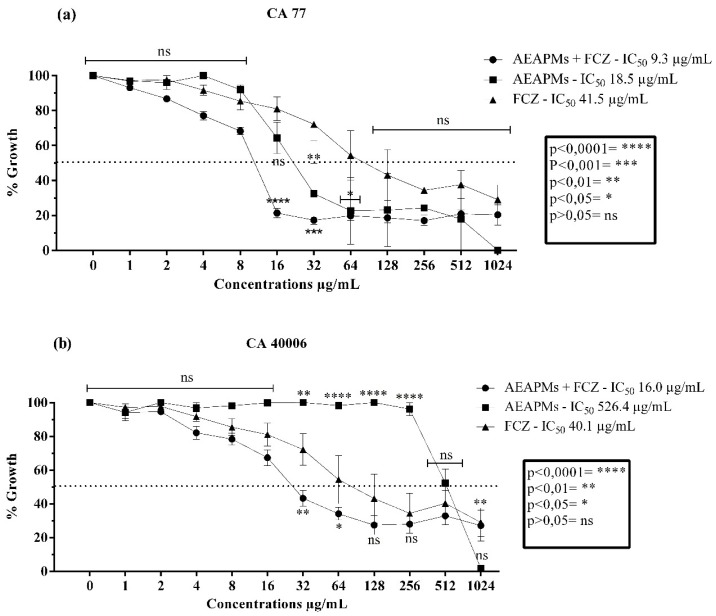
Cell viability curves and IC_50_ values (concentration responsible for 50% colony growth inhibition) of the *Mesosphaerum suaveolens* aqueous aerial parts extract (AEAPMs) and fluconazole (FCZ) in μg/mL against *Candida albicans*. (**a**) Antifungal activity against strains of *Candida albicans* LM 77. (**b**) Antifungal activity against strains of *Candida albicans* INCQS 77.

The aerial parts extract was able to potentiate the effect of fluconazole more effectively than the leaf extract (Figure 3). Such an effect was significant from the 16 µg/mL concentration for CA LM 77 (Figure 3a) and 32 µg/mL for CA INCQS 40006 (Figure 3b). Similar to the leaf extract, the AEAPMs presented antifungal effects at clinically relevant concentrations and were able to inhibit 50% of fungal growth at 18.5 µg/mL (CA LM 77) and 526.4 µg/mL (CA INCQS 40006) concentrations. It is noteworthy that *C. albicans* fungal growth was completely inhibited at the highest concentration of 1024 µg/mL.

**Figure 4:**
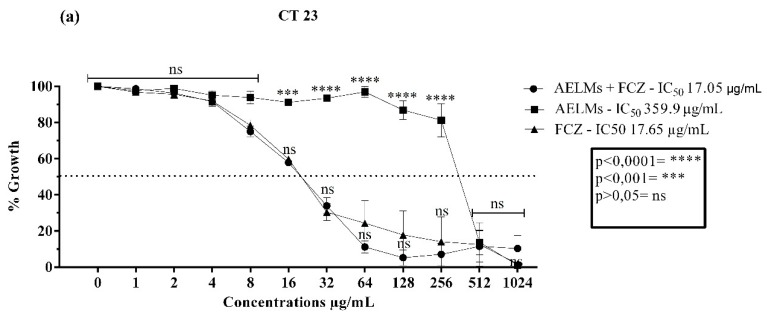

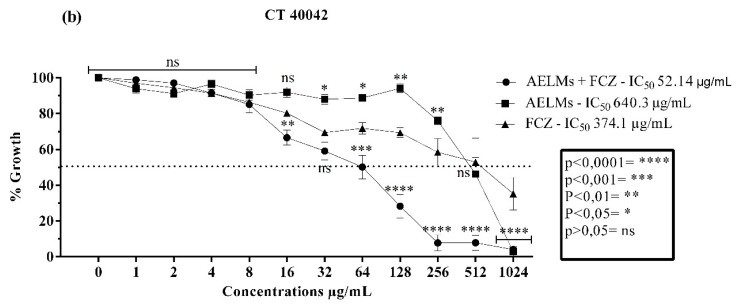
Cell viability curves and IC_50_ values (concentration responsible for 50% colony growth inhibition) of the *Mesosphaerum suaveolens* aqueous leaf extract (AELMs) and fluconazole (FCZ) in μg/mL against *Candida tropicalis*. (**a**) Antifungal activity against strains of *Candida tropicalis* LM 23. (**b**) Antifungal activity against strains of *Candida tropicalis* INCQS 40042.

**Figure 5:**
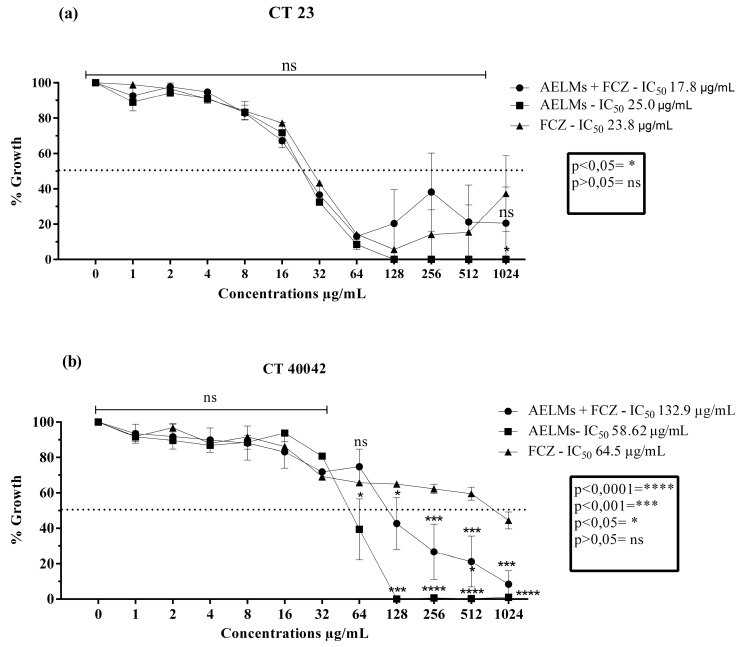
Cell viability curve and IC_50_ values (concentration responsible for 50% colony growth inhibition) of the *Mesosphaerum suaveolens* aqueous aerial parts extract (AEAPMs) and fluconazole (FCZ) in μg/mL against *Candida tropicalis*. (**a**) Antifungal activity against strains of *Candida tropicalis* LM 23. (**b**) Antifungal activity against strains of *Candida tropicalis* INCQS 40042.

The leaf extract as well as the aerial parts extract presented clinically relevant activities for *C. tropicalis* strains. For the *C. tropicalis* LM 23 strain, the leaf extract obtained an IC_50_ of 359.9 µg/mL (Figure 4a) and the aerial parts extract an IC_50_ of 25 µg/mL (Figure 5a). Neither extract presented a significant action over fluconazole modulation. For CT INCQS 40042, the leaf extract significantly modulated the antibiotic used, reducing its IC_50_ (Figure 4b). However, the aerial parts extract presented an antagonistic effect, that is, it negatively affected the effect of the antibiotic, since it obtained an IC_50_ of 64.5 µg/mL, increasing this parameter by more than 100% (Figure 5b).

## 3. Discussion

In ethnobotanical studies, *M. suaveolens* has been reported to treat certain disorders such as gastrointestinal tract disorders [34,35]. The use of *M. suaveolens* in folk medicine is justified, since the species has chemical compounds capable of decreasing the proliferation of *Candida* strains. It is worth noting that among these substances, phenolics, such as catechin and apigenin, are highly active in different biological properties, suggesting a possible synergistic effect [36,37,38]

Mbatchou et al. [39], evaluating the action of the aqueous extract of the *M. suaveolens* aerial parts, also showed that the species presents antifungal action by halo inhibition against *C. albicans* strains when compared with the antibiotic griseofulvin. Such biological activity is due to the phytochemical constituents found in the species such as alkaloids, flavones, flavonols, flavonones, terpenoids, tannins, aldehydes, and ketones.

The species is aromatic and produces volatile terpenes known as essential oils which have several biological activities, such as antimicrobial, helping in the protection of vegetables. Thus, the species’ essential oil is widely studied from the phytochemical and antifungal points of view [22]. For example, a study by Bachheti et al. [31] demonstrated that this plant’s essential oil exhibits relevant activity against *C. albicans* and *C. tropicalis* at different concentrations. Malele et al. [30] also reported a strong antifungal activity for this plant species by showing significant inhibitory effects against different fungi, including *C. albicans*.

The drug used in this research belongs to the azole class, so the antibiotic acts by inhibiting the enzyme lanosterol 14-α-steroldemethylase. In *Candida* strains, these enzymes are encoded by the ERG11 genes and have as their substrate the lanosterol involved in ergosterol synthesis, which is the main cell membrane compost. Thus, antibiotics of such a current class block ergosterol synthesis in order to inhibit yeast growth through accumulation of 14-α-methyl sterols [40]. Although fluconazole is effective in combating candidiasis, strains have gained resistance over the years due to the fact of prolonged treatments [41].

Alternatively, some products are used to modulate, in other words, enhance the effectiveness of antibiotics and may be natural or synthetic products [42,43,44]. In our study, we can see that *M. suaveolens* extracts were able to modulate the effect of the drug against strains of *C. albicans* and *C. tropicalis*, so that the species has compounds that are antifungal agents. For Spitzer et al. [45], various combinations of drugs were assessed and indicated that some compound classes cause synergistic membrane permeability or inhibit sphingolipid biosynthesis. This indicates that certain compounds have the ability to potentiate the action of first line drugs, thus corroborating our results and which obtained significance with respect to their combined activity with fluconazole.

It was observed that the biological activity was more expressive for the leaves extract. Such an effect is explained by the fact that the leaves are the parts that are most involved with the production of secondary metabolites. Since photosynthesis occurs in the leaf limb, consequently, it happens in the production of terpene precursors and phenolic compounds. Associated with this factor is biochemical co-evolution, in which, because leaves are the parts consumed by herbivores, they produce and concentrate biologically active phytochemicals [46,47]. Thus, EALMs have higher antifungal activity.

The analyses of HPLC showed that the species is rich in flavonoids, and such constituents are capable of disrupting fungal membranes [48,49] and may inhibit the budding process and decrease the Ca^+^ and H^+^ homeostasis [50]. In the present study, caffeic acid and quercetin were the major compounds present in both extracts of *M. suaveolens* with caffeic acid standing out, as it is always present in the chemical composition of *M. suaveolens* extracts [22,51]. Such compounds may be associated with reduced yeast growth, as Lima et al. [52] report that caffeic acid (3,4-dihydroxy cinnamic acid) exerts significant microbiological activity and that its mechanism of action is directly related to the inhibition of the RNA polymerase enzyme. According to Buffalo [53], caffeic acid may act at a molecular level causing microorganismal death or inhibiting their reproduction, and it may also interact directly with cellular components causing irreversible damage to these fungi. In addition to these mechanisms of action, caffeic acid may inhibit the isocitrate lyase (ICL1) involving the glyoxylate cycle in *C. albicans* [54].

Referring to quercetin, this flavonoid has the ability to induce cell death by apoptosis of *C. albicans* strains, explaining the fungicidal effect at concentrations of 1024 μg/mL. Such a compound is capable of acting in several ways such as inhibition of biofilm formation, hyphae development, phospholipase, proteinase, and esterase [54]. Molecular anchor studies have shown strong molecular interactions between quercetin and adenylate cyclase of ATP binding bag through the formation of hydrogen bonds and hydrophobic and ionic interactions with the important residues of the adenylate cyclase ATP binding bag (Gln969, Thr1105, Ser1108, Arg1109, Asn1110 and Gly1061) thus inhibiting the pathogenicity of *C. albicans*. Such an enzyme plays roles in regulating drug resistance [55,56]. In addition to this species, quercetin has significant intrinsic synergistic activity with fluconazole against *C. tropicalis* strains by promoting apoptosis by phosphatidylserine exposure in the plasma membrane and morphological changes, mitochondrial depolarization, intracellular ROS accumulation, condensation, and fragmentation of DNA [57].

## 4. Materials and Methods

### 4.1. Plant Collection

**Figure 6:**
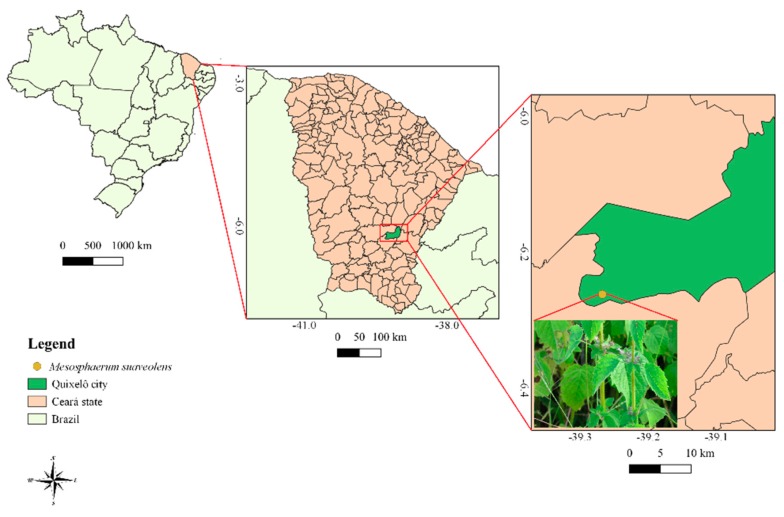
Map of the *Mesosphaerum suaveolens* species collection in the Municipality of Quixelô, CE, Brazil.

The *M. suaveolens* botanical material was collected in March of 2016 at a south-central region in Ceará-Brazil, municipality of Quixelô, under the following coordinates: latitude (−6°15’43.0056’), longitude (−39°16’2.5926”), 193.2 m above sea level (Figure 6). A sample specimen of the plant species was selected and identified by José Weverton Almeida Bezerra and was deposited in the Herbarium Caririense Dárdano de Andrade-Lima-HCDAL/URCA under the number #12,104.

### 4.2. Preparation of the *Mesosphaerum Suaveolens* Extracts

The *M. suaveolens* leaves (320 g) and aerial parts (leaf, stem, flower) (270 g) were collected and washed in running water to remove possible traces of impurities before being left to dry. Subsequently, the extracts were properly packaged in autoclaved glass vials and 1 L of distilled boiling water was added to the vials. After water immersion over a period of 72 h, the solid portion of the liquid part was double filtrated over cotton and subsequently placed in a properly sanitized bottle to be frozen.

After freezing, the extracts were lyophilized to remove any remaining water in order to obtain the crude extract. The *M. suaveolens* aqueous leaf extract (AELMs) had a yield of 2.65%, while the *M. suaveolens* aqueous aerial parts extract (AEAPMs) had a yield of 3.77%.

### 4.3. Quantification of Compounds by HPLC-DAD

*Mesosphaerum suaveolens* extracts were injected onto reversed phase Phenomenex C_18_ columns (4.6 mm × 250 mm) packed with 5 μm diameter particles. The mobile phases A and B contained Milli-Q water, acidified to a pH of 2.0 with 1% acetic acid (A) and methanol (B). Correspondingly, the solvent gradient was used as follows: 0–10 min, 5% B; 10–25 min, 15% B; 25–40 min, 30% B; 40–55 min 50% B; 50–65 min 70% B; 65–80 min, 100% B, following the method described by Waczuk et al. [58] with slight modifications. The *M. suaveolens* extracts were analyzed at a 12 mg/mL concentration, with a flow rate of 0.6 mL/min and injection volume of 40 μL. The sample and mobile phases were filtered through 0.45 μm membrane filter (Millipore) and then degassed by ultrasonic bath prior to use. Standard reference stock solutions were prepared in methanol:water (1:1, v/v) at concentrations ranging from 0.030–0.500 mg/mL. Quantifications were carried out by peak integration using the external standard method at 254 nm for gallic acid and ellagic acid, 280 nm for catechin, 327 nm for caffeic acid and chlorogenic acid, and 366 nm for quercetin, apigenin, and rutin. The chromatography peaks were confirmed by comparing their retention times with those of reference standards and by DAD spectra (200 to 700 nm). All chromatography operations were carried out at ambient temperature and in triplicates.

### 4.4. Antifungal Activity

#### 4.4.1. Fungal Strains and Culture Media Used

Two standard yeast strains were used (i.e., *Candida albicans* (CA LM 77) and *Candida tropicalis* (CT LM 23)) as well as two clinical isolates (i.e., *C. albicans* (CA INCQS 40006) and *C. tropicalis* (CT INCQS 40042)). The used strains were obtained from the National Institute for Quality Control in Health (INCQS). After incubation in Sabouraud Dextrose Agar (SDA, KASVI) for 24 h at 37 °C, aliquots were removed and transferred to test tubes containing 3 mL of sterile saline (0.9%). A comparison with a McFarland scale was used to determine if the concentration of the inoculum was standardized by comparing its turbidity with the 0.5 standard, giving a yeast suspension of 1 × 10^5^ cells/mL [59].

#### 4.4.2. Drugs, Reagents, and Solution Preparation

Fluconazole (Prati, Donaduzzi & Cia Ltd.a., Toledo, Brazil) was used as the reference antifungal drug. All solutions were prepared following the recommendations of the Clinical and Laboratory Standards Institute (CLSI) [60]. Fluconazole was diluted in water to achieve an initial concentration of 2.048 µg/mL, while the extracts were diluted in distilled water.

#### 4.4.3. IC_50_ Determination and Cellular Viability Curve

The IC_50_ was determined in Sabouraud Dextrose Broth (Difco Ltd.) by the microdilution method using ELISA plates. Briefly, 100 µL of culture medium fungal strains (10^5^ CFU/mL, 10%) were distributed in 96 well plates followed by a serial dilution with 100 µL of varying concentrations (from 1024 to 1 µg/mL) of the extracts. The assays were performed concomitantly with the standard antifungal. The plates were read spectrophotometrically at 630 nm using a microplate reader to determine cellular viability [59].

#### 4.4.4. Modulatory Effect of Extracts from *M. Suaveolens*

To evaluate the effect of the extracts over fluconazole activity, the extracts were tested at sub-inhibitory concentrations (MIC/8). Eppendorfs containing SDA (Sabouraud Dextrose Agar) culture medium, 10% inoculum, and the extracts were prepared. A 100 µL solution containing the culture medium, the inoculum (10%), and the extracts was distributed in alphabetical order in each well of an ELISA plate up until the penultimate cavity. For the test, a dilution control for fluconazole, a dilution control for the modulation, and a sterility control of the culture medium were performed. The plates were read spectrophotometrically at 630 nm using a microplate reader to determine cellular viability [40,59].

### 4.5. Statistical Analysis

Data are expressed as the mean ± SEM of at least three independent experiments. Differences among the quantities of identified compounds were analyzed using a one-way analysis of variance (ANOVA) following by Tukey’s test. The microbiological results were analyzed by a one-way ANOVA followed by Bonferroni’s post-hoc test to evaluate the differences among the experimental groups. Significance was considered at *p* < 0.05.

## 5. Conclusions

The popular use of *M. suaveolens* to treat diseases, such as gastrointestinal tract disorders, is related to its secondary metabolite chemical heterogeneity which includes phenolic substances. The species presents a high potential for the formulation of drugs to combat infections caused by strains from the *Candida* genus in addition to potentiating the effect of fluconazole.

## Figures and Tables

**Table 1:** Chemical composition of the *Mesosphaerum suaveolens* extracts by HPLC-DAD.

Compounds	AELMs	AEAPMs
	mg/g	mg/g
Catechin	6.05 ± 0.01 b	-
Chlorogenic acid	-	3.89 ± 0.01 a
Caffeic acid	13.27 ± 0.02 c	14.25 ± 0.04 b
Ellagic acid	5.98 ± 0.03 b	8.01 ± 0.02 c
Rutin	2.81 ± 0.01 a	-
Quercetin	7.03 ± 0.01 d	14.70 ± 0.01 b
Apigenin	-	4.11 ± 0.01 a

Results are the mean ± standard deviation (SD) from three determinations. Averages followed by different letters differ by Tukey’s test at *p* < 0.05.
